# A diagnostic challenge: Life-threatening drug reaction with eosinophilia and systemic symptoms in the setting of a SARS-CoV-2 and human herpesvirus-6 reactivation in a patient with severe hepatic injury on bupropion

**DOI:** 10.1016/j.jdcr.2023.07.025

**Published:** 2023-08-01

**Authors:** Djoni Elkady, Chiamaka L. Okorie, Blanca Estupiñan, Mitul B. Modi, Scott J. Cotler, Eden Lake

**Affiliations:** a Loyola University Chicago Stritch School of Medicine, Maywood, Illinois; b Geisel School of Medicine, Dartmouth College, Lebanon, New Hampshire; c Division of Dermatology, Loyola University Medical Center, Maywood, Illinois; d Department of Pathology and Laboratory Medicine, Loyola University Medical Center, Maywood, Illinois; e Division of Hepatology, Loyola University Medical Center, Maywood, Illinois

**Keywords:** bupropion, COVID, DILI, DRESS, drug-induced, drug reaction, hypersensitivity syndrome, SARS-CoV-2

## Introduction

Drug reaction with eosinophilia and systemic symptoms (DRESS) is a severe cutaneous adverse reaction typically characterized by fever, rash, lymphadenopathy, eosinophilia, and multiorgan involvement. Common offending agents include anticonvulsants, antidepressants, and sulfonamides among others.^1^ The pathogenesis is thought to be secondary to toxic drug metabolite accumulation because of enzyme deficiencies. DRESS typically exhibits a delayed onset with symptoms starting 2 to 8 weeks following drug exposure. Morbilliform skin eruption is present in 80% of cases, and the liver and kidneys are frequently affected.^1^^,^^2^

We report an atypical case of DRESS with overlapping features of drug-induced liver injury (DILI) from bupropion in a patient who was critically ill, recovering from SARS-CoV-2 infection with human herpesvirus type 6 (HHV-6) reactivation. This case underscores the diagnostic complexity of DRESS, its association with DILI, and the influence of SARS-CoV-2 and HHV-6 on disease severity.

## Case presentation

**Fig 1:**
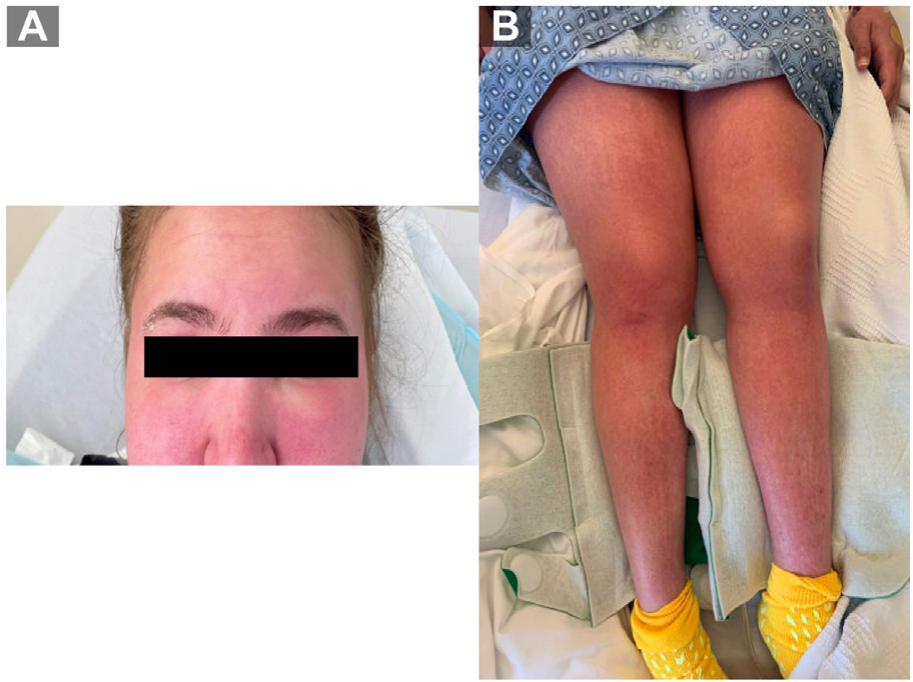
Patient with a diffuse morbilliform rash involving the face with edema (**A**), arms, trunk, back, and legs (**B**).

A 23-year-old woman with a history of leukocytoclastic vasculitis and depression presented with a 2-week history of fever, cough, congestion, and a generalized pruritic cutaneous eruption. Physical examination revealed diffuse erythematous papules that coalesced on her face, trunk, and bilateral upper and lower extremities, accompanied by facial, periorbital, and bilateral lower extremity edema (Fig 1). Cervical, axillary, and inguinal lymphadenopathy was noted. She had a fever (39.5 °C) and tachycardia. She denied recent travel or illicit drug use but had been taking bupropion for depression for 5 weeks before admission. Two weeks before admission, she experienced flu-like symptoms, which she attributed to a SARS-CoV-2 infection, and the cutaneous findings appeared 5 days later. Laboratory test results showed elevated aminotransferase levels, eosinophilia, leukocytosis, and a positive SARS-CoV-2 test result. A chest radiograph demonstrated increased interstitial markings and opacities at the lung bases. DRESS was highly suspected with a Registry of Severe Cutaneous Adverse Reaction (RegiSCAR) score of 8 (Table I), with consideration for SARS-CoV-2 viral exanthem and DILI.

**Table I:** Results of the Registry of Severe Cutaneous Adverse Reaction scoring system used to diagnose drug reaction with eosinophilia and systemic symptoms^1^

Clinical parameters	Score				Patient score	Clinical findings (normal value)
	−1	0	1	2		
Fever ≥101.3 °F (38.5 °C)			Yes		1	
Lymphadenopathy∗			Yes		1	Cervical, axillary, and inguinal lymphadenopathy
Eosinophilia†				Yes	2	Eosinophil: 2.1 × 10^3^ (0-0.7 K/mm^3^)
Atypical lymphocytes		No			0	WBC: 19.7 (3.5-10.5 K/UL)
Skin rash suggestive of DRESS‡			Yes		1	Facial and periorbital edema, desquamation
Skin rash ≥50% of body surface area			Yes		1	
Skin biopsy suggestive of DRESS		Yes			0	
Liver involvement			Yes		1	AST, 3567 (10-40 u/L) ALT, 2275 (7-35 u/L)
Disease duration ≥15 d		Yes			0	
Exclusion of other causes§			Yes		1	Hepatitis A, B, C, antinuclear antibody, blood cultures negative
Total score					8	

Total score < 2: Excluded, 2-3: Possible, 4-5: Probable, ≥ 6: Definite.

*ALT*, alanine aminotransferase; *AST*, aspartate aminotransferase; *WBC*, white blood cells.

∗ Lymphadenopathy >1cm, at least 2 sites.

† Eosinophils 0.7-1.49 × 10^3^ K/mm^3^ = 1; ≥1.5 × 10^3^ K/mm^3^ = 2.

‡ Suggestive features: ≥2 facial edemas, purpura, infiltration, and desquamation.

§ Score 1 point if 3 of the following tests are performed and are negative: Hepatitis A, B, and C, mycoplasma, chlamydia, antinuclear antibody, and blood culture.

**Fig 2:**
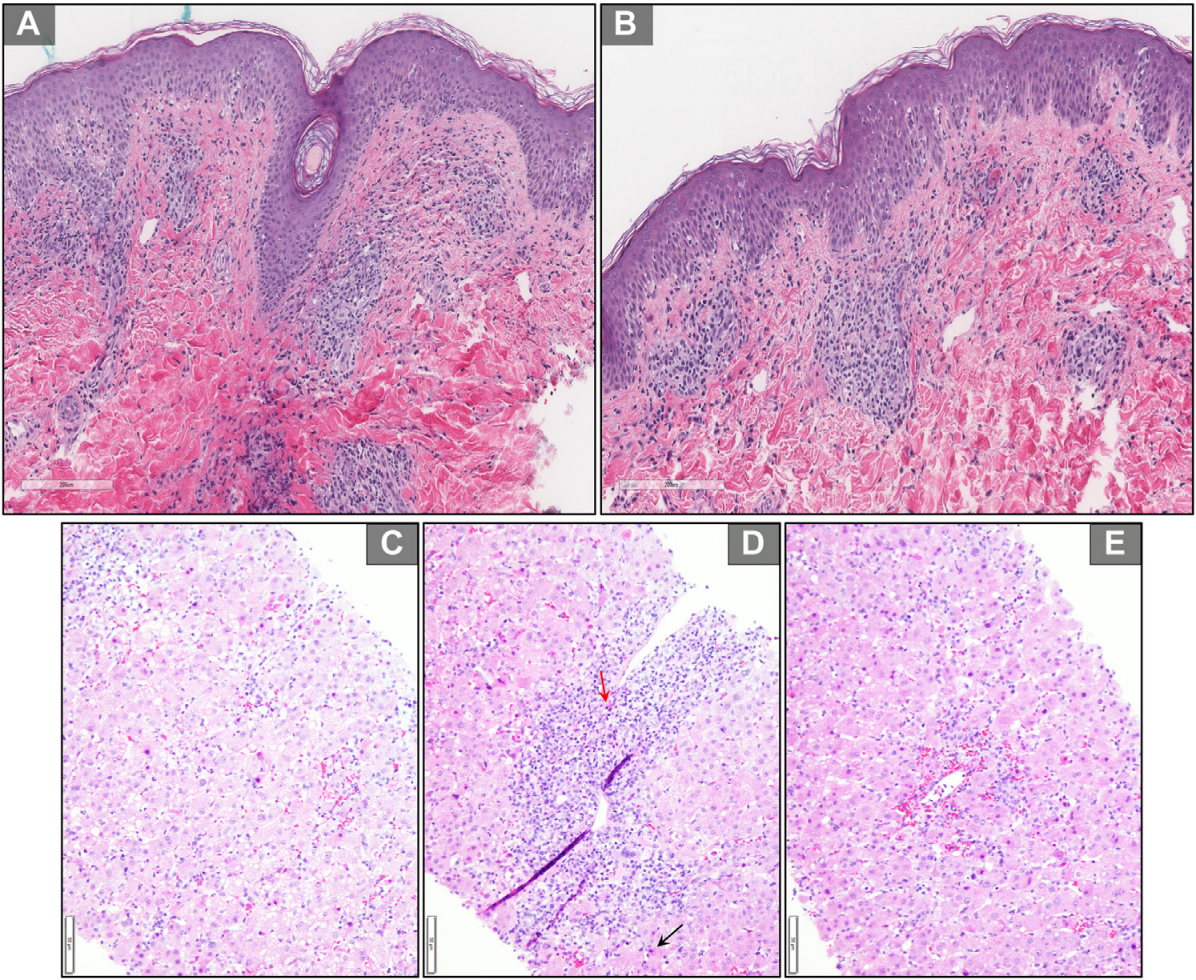
(**A**) 200-um scanning magnification of hematoxylin and eosin skin biopsy demonstrating basket-weave orthokeratosis with interface dermatitis and vacuolar interface changes, (**B**) superficial perivascular and interstitial mixed inflammation with lymphocytes as well as scattered neutrophils and eosinophils, associated scattered apoptotic keratinocytes, (**C**) 50-um scanning magnification of hematoxylin and eosin liver biopsy demonstrating lobular injury with hepatocyte swelling and numerous acidophilic bodies, (**D**) mixed inflammation with predominant small mature lymphocyte, rare plasma cell (*black arrow*) and eosinophil (*red arrow*) infiltrates as well as focal interface activity, and (**E**) perivenulitis.

Bupropion was discontinued because of suspected drug hypersensitivity, and prednisone 60 mg (1 mg/kg) was initiated. Cultures (blood, urine, and stool) and urine drug screen were negative. Alpha-1 antitrypsin, ceruloplasmin and antinuclear, antineutrophil cytoplasmic, antimitochondrial, and smooth muscle antibodies were normal. Viral serologies for cytomegalovirus, Epstein–Barr virus, parvovirus B19, herpes simplex virus, adenovirus, hepatitis A, B, and C were negative. Polymerase chain reaction test was positive for HHV-6 (>2,000,000 copies/mL). Abdominal ultrasound showed mild hepatomegaly with a “starry sky” appearance of the liver parenchyma, suggesting acute hepatitis. Skin biopsy of the left thigh displayed basket-weave orthokeratosis with interface dermatitis and mixed interstitial inflammation with lymphocytes, neutrophils, and eosinophils, suggestive of drug eruption (Fig 2).

After initial improvement, she developed intermittent fevers, tachycardia, scleral icterus, and asterixis. Prednisone was increased to 120 mg (2 mg/kg) on day 7. Despite escalating steroid therapy, liver enzymes continued to uptrend to maximum values (aspartate aminotransferase, 3567 u/L; alanine aminotransferase, 2275 u/L; alkaline phosphatase, 440 u/L; and total bilirubin, 10.6 mg/dL). Model for end-stage liver disease score reached 28, prompting liver transplant evaluation. A liver biopsy revealed lobular injury and portal inflammation with predominant mature lymphocytes, rare plasma cells, and eosinophils (Fig 2). These findings suggested DILI or acute autoimmune hepatitis (AIH). She was transitioned to intravenous methylprednisolone 125 mg. Her vital signs, cutaneous findings, and liver enzymes improved. She was discharged on a prednisone taper with close hepatology and dermatology follow-up.

## Discussion

Cutaneous manifestations of DRESS include a morbilliform eruption, often with facial edema, scaling, and/or purpura. The liver is the most common internal organ affected.^1^ Involvement is generally mild, but acute liver failure has been reported.^2^ Diagnosing DRESS can be challenging. It should be suspected in a patient who received a new high-risk medication 2 to 8 weeks before presentation with characteristic symptoms. The RegiSCAR scoring system provides objective diagnostic criterion, but there is overlap with other disease processes, such as infectious and inflammatory diagnoses.^1^^,^^3^ Our patient had a RegiSCAR score of 8 supporting a “definite” diagnosis of DRESS (Table I).

DILI and AIH were possible etiologies of the patient’s liver findings. However, relapse after steroid withdrawal is more likely with AIH.^4^ Our patient’s liver enzymes remained consistently normal during the 6-month follow-up period after steroid discontinuation. Of note, our patient had a Roussel Uclaf Causality Assessment Method score of 7 suggesting DILI as a “probable” cause (Table II).^5^ Case reports of bupropion-induced liver injury have been documented, and liver injury in DRESS might be one variant of DILI as evidenced by our patient’s course.^6^^,^^7^

**Table II:** Results of the Roussel Uclaf Causality Assessment Method score to assess the likelihood of drug-induced liver injury in a mixed type pattern^5^

Criteria	Description	Score	Patient score
Time to onset of reaction	**5-90 d of drug start** < 5 or > 90 d of drug start ≤30 d from drug cessation	+2 +1 +1	+2
Course of ALP after drug cessation (percentage difference between ALP peak and ULN)	**Decrease ≥ 50% in 180 d** Decrease < 50% in 180 d Persistence, increase or no information	+2 +1 0	+2
Risk factors	Age ≥ 55 Alcohol use or pregnancy	+1 +1	0
Concomitant drug(s)	**None or no information** Time to onset incompatible Time to onset compatible but unknown reaction Time to onset compatible and known reaction Role proved in this case (positive rechallenge or validated test)	0 0 −1 −2 −3	0
Exclusion of other causes of liver injury	**All group I**∗**and II**†**ruled out** All of group I ruled out 4-5 of group I ruled out <4 of group I ruled out Nondrug causes highly probable	+2 +1 0 −2 −3	+2
Previous hepatotoxicity of drug	Reaction unknown **Reaction published but unlabeled** Reaction labeled in product characteristics	0 +1 +2	+1
Response to rechallenge	Positive Compatible Negative **Not performed or not interpretable**	+3 +1 −2 0	0
Total points			7

Total score ≤ 0: Excluded, 1-2: Unlikely, 3-5: Possible, 6-8: Probable, ≥ 9: Highly probable.

*ALP*, alkaline phosphatase; *ULN*, upper limit of normal.

∗ Group I causes: acute viral hepatitis A or B or C or E, biliary obstruction, alcoholism, and recent hypotension (shock liver).

† Group II causes: complication of underlying disease(s), clinical and/or biologic context suggesting cytomegalovirus, Epstein–Barr virus, or herpes simplex virus infection.

HHV-6 reactivation has been associated with DRESS.^8^ Systemic corticosteroids have proven helpful in controlling the immune response in DRESS; however, corticosteroids can also exacerbate HHV reactivations and, if tapered rapidly or underdosed, can lead to organ failure secondary to an uncontrolled immune response.^8^ Despite concurrent reactivation of HHV-6, increasing our patient's corticosteroid dosage improved liver function. Tohyama et al^9^ analyzed the effects of corticosteroids on viral reactivation in DRESS and found that the use of high-dose corticosteroids within 1 week of disease onset was associated with lower rates of HHV-6 reactivation.^9^ In contrast, low-dose or delayed initiation of corticosteroids were not found to suppress HHV-6 occurrence and viremia. These findings suggest that timely high-dose corticosteroids may limit the proliferative effects of HHV-6 reactivation, which may have contributed to the recovery of our patient’. Further research is needed to optimize systemic corticosteroid therapy for suppressing HHV-6 reactivation in DRESS.

Our patient's concurrent SARS-CoV-2 infection adds complexity to their presentation because SARS-CoV-2 can exhibit a cytokine storm resembling the immunologic response seen in DRESS. ^10^ It has been suggested that SARS-CoV-2 may enhance abnormal lymphocyte and eosinophil responses in the setting of DRESS.^10^ Additionally, HHV-6 reactivation has been observed in patients with SARS-CoV-2, linking to a shared mechanism and inflammatory response observed in DRESS. Although SARS-CoV-2 is unlikely to be the sole cause of our patient's organ dysfunction, considering the suspected drug, its hepatotoxicity, and the temporal relationship with DRESS symptoms, the shared immunologic response triggered by SARS-CoV-2 may contribute to the exacerbation of DRESS manifestation.

The current case highlights the diagnostic challenges of DRESS and the need for further research to understand interactions among SARS-CoV-2, drug reactions, and viral reactivations. In light of the overlapping features between DRESS and DILI, it is important to report unusual manifestations to aid in diagnosis and the management of complex cases. An interdisciplinary approach involving dermatologists, hepatologists, and other relevant specialists is strongly advocated to facilitate case comparison and accurate phenotyping.

## Conflicts of interest

None.
